# Supplementary material for: Sphingoid long chain bases prevent lung infection by Pseudomonas aeruginosa
Source: EMBO Mol Med. 2014 Aug 1;6(9):1205–14. doi: 10.15252/emmm.201404075 (PMC4197866; doi:10.15252/emmm.201404075)
Supplement: Supplementary file 1 — Supplementary Information [file emmm0006-1205-SD1.pdf]

Supplementary information

Table of contents

1. Specificity of the anti-SPH antibody
2. Incubation of bacteria with ceramide and acid ceramidase
3. Supplementary Figure 1. The anti-sphingosine antibody does not recognize sphinganine.
4. Supplementary Figure 2. Trachea and bronchi from Cers2 null mice accumulate ceramide.
5. Supplementary Figure 3. Inhalation of acid ceramidase, SPH or FTY720 prevents the clinical symptoms of *P. aeruginosa* pneumonia
6. Supplementary Figure 4. Inhalation of acid ceramidase, SPH or FTY720 prevents cytokine release after infection with *P. aeruginosa*.
7. Supplementary Figure 5. Inhalation of acid ceramidase, sphingosine or FTY720 prevents leukocyte influx into the lung upon infection with *P. aeruginosa*
8. Supplementary Figure 6. Inhalation of acid ceramidase, sphingosine or FTY720 does not induce inflammation.
9. Supplementary Figure 7. In situ enzyme kinase assay with Mg^{2+}

Specificity of the anti-SPH antibody

Several lines of evidence are provided to demonstrate the specificity of the anti-sphingosine antibody. (i) The company (Alfresa Pharma Corporation, Japan) excluded binding of the antibody to ceramide, sphingosine 1-phosphate, sphingomyelin, cerebroside, phosphatidylserine, lyso-phosphatidylethanolamine and lyso-phosphatidylcholine (http://search.cosmobio.co.jp/cosmo_search_p/search_gate2/docs/ALF_/274042010.20090826.pdf). (ii) The reduction of staining intensity of the antibody in CF and CerS2 null mice, compared to WT mice, excludes the possibility that the antibody detects lipids that are found at normal levels, or are elevated in CF and CerS2 null mice (such as sphingomyelin, ceramide, sphingosine 1-phosphate and gangliosides). (iii) Inhalation of CF and CerS2 null mice, or *in vitro* treatment of isolated, but intact trachea with AC, resulted in an increase in immunofluorescence using the anti-sphingosine antibody (see Figure 1). The increased staining correlated with the increase of sphingosine levels determined by *in situ* kinase assays on AC-inhaled mice or on isolated trachea (Figure 2). (iv) CF mice trachea were incubated *in vitro* with dihydrosphingosine (sphinganine), fixed and the sections stained with the anti-sphingosine antibody (Supplementary Figure 1, *upper panel*). The anti-sphingosine antibody did not bind to sphinganine as indicated by the very similar labeling intensity of untreated or sphinganine-treated CF trachea. Controls that measured the total content of sphinganine after organic extraction of lipids followed by phosphorylation of sphingosine or sphinganine *in vitro* with [^{32}P]γATP, confirmed that sphinganine was taken up by the trachea (not shown). (v) We tested binding of the anti-sphingosine antibody to sphingosine or sphinganine by incubation of 200 pmol sphingosine or sphinganine (in 125 mM NaCl, 25 mM TrisHCl (pH 7.4), 10 mM EDTA, 10 mM sodium pyrophosphate, 3% Nonidet P40 and 10 µg/ml aprotinin and leupeptin) with 2.5 µg of the anti-sphingosine antibody for 45 min at 4°C. Immuno-complexes were immobilized with L-agarose, washed 5 times in the same buffer, the beads pelleted, the supernatants discarded and the beads extracted in 200 µl H_2O followed by 600 µl $CHCl_3$/CH_3OH/1N HCl (100:200:1, v/v/v). The organic phase was collected, dried, resuspended in 20 µL of a detergent solution (7.5% [w/v] n-octyl glucopyranoside, 5 mM cardiolipin in 1 mM diethylenetriaminepentaacetic acid [DTPA]), and sonicated in a water bath for 10 min. The kinase reaction was initiated by addition of 70 µL of a reaction mixture containing 0.001 units sphingosine kinase in 50 mM HEPES (pH 7.4), 250 mM NaCl, 30 mM $MgCl_2$ 1 mM adenosine triphosphate (ATP) and 10 µCi [^{32}P]γATP. The kinase reaction was terminated after 45 min and samples were further extracted and processed as described in Methods. The results (Supplementary Figure 1, *lower panel*) indicate that the antibody does

not immunoprecipitate sphinganine. Controls in which sphinganine or sphingosine were incubated directly with sphingosine kinase demonstrate that sphingosine kinase phosphorylates both lipids and that both lipids run very similar on the TLC plate.

Incubation of bacteria with ceramide and acid ceramidase

Incubation of the bacteria for 120 min with 2 μM ceramide in the presence of 100 units AC in a volume of 500 μl resulted in the release of 400 pmol/500 μl sphingosine and killing of 62 ± 9.6 % killing of *P. aeruginosa.* Ceramide added together with heat-inactivated AC (95°C for 10 min) was without effect.

Supplementary Figures

Supplementary Figure S1. The anti-sphingosine antibody does not recognize sphinganine. *Upper panel*, CF or WT mice trachea were incubated *in vitro* with dihydrosphingosine (sphinganine), fixed and stained with the anti-sphingosine antibody. *Lower panel*, a representative autoradiogram of a TLC plate from 4 independent experiments indicating that the antibody does not immunoprecipitate sphinganine. Data are representative for 5 (upper panel) or 4 (lower panel) independent experiments.

Supplementary Figure S2. Trachea and bronchi from Cers2 null mice accumulate ceramide.

Staining of paraffin sections from trachea and bronchi of CerS2 null mice with a Cy3-coupled anti-ceramide antibody reveals accumulation of ceramide in bronchial and tracheal epithelial cells of CerS2 null mice. Representative images are shown. Data are means ± s.d. of fluorescence levels (in arbitrary units, a.u.). n=3. Numbers above bars indicate the exact calculated p-values.

Supplementary Figure S3. Inhalation of acid ceramidase, SPH or FTY720 prevents the clinical symptoms of *P. aeruginosa* pneumonia

Overall sickness score after intranasal infection with 1×10^8 CFU of *P. aeruginosa* strains 762 (A), PA14 or ATCC 27853 (B). Mice were inhaled with 0.9% NaCl or AC 1 h prior to infection (before infection, b.i.), or with SPH or FTY720 1 h before or 1 h after (after infection, a.i.) infection. Sickness score 4, very severely affected (unresponsive animal, heavy breathing, low body temperature, ruffled fur); score 3, severely affected (ruffled fur, heavy breathing, lower body temperature); score 2, moderately affected (ruffled fur, breathing slightly impaired, normal body temperature), score 1, slightly affected (ruffled fur); score 0, unaffected (healthy appearance). Data are means ± s.d., n=4. Numbers above bars indicate the exact calculated p-values.

Supplementary Figure S4. Inhalation of acid ceramidase, SPH or FTY720 prevents cytokine release after infection with *P. aeruginosa*.

TNF-α was determined by ELISA in lungs from mice that were infected with the indicated *P. aeruginosa* strain or left uninfected. Shown are mean ± s.d., n=3. Numbers above bars indicate the exact calculated p-values.

Supplementary Figure S5. Inhalation of acid ceramidase, sphingosine or FTY720 prevents leukocyte influx into the lung upon infection with *P. aeruginosa*

Influx of leukocytes into the lung after infection and the indicated treatment or inhalation of the drugs only was determined by hemalaun staining (A-D) or immunostaining with Cy3-coupled anti-Gr-1 antibodies (E-H). Mice were infected with *P. aeruginosa* 762 or ATCC 27853 for 4 h, the lungs removed, stained and analyzed by light or fluorescence microcopy. Shown are representative figures from 3 independent experiments.

Supplementary Figure S6. Inhalation of acid ceramidase, sphingosine or FTY720 does not induce inflammation.

Mice were inhaled with AC, SPH or FTY720 and cytokines or the influx of leukocytes determined by ELISA of lung homgenates or hemalaun stainings of lung sections, respectively, were measured to determine whether the drugs alone (without any infection) induce any inflammation. For untreated samples and studies 4 h after inhalation, see images in Fig 3F, G and supplementary Fig S4C, D. Shown are the mean ± SD. Numbers above bars indicate the exact calculated p-values.

Supplementary Figure S7. In situ enzyme kinase assay with Mg^{2+}

The airway liquid contains Mg^{2+} at 1.9 mmol/liter (Baconnais *et al* 1999). Since we added a very small volume in all *in situ* kinase assays, we left the Mg^{2+}-concentration high enough to perform the *in situ* kinase assays. Thus, in an attempt to leave the airway surface liquid unaltered as much as possible, we did not add any further Mg^{2+}. However, we also performed controls in which we added $MgPO_4$. In these experiments the surface SPH was determined as described in Methods by application of sphingosine kinase in a solution of in 150 mM sodium acetate (pH 7.4), 30 mM $MgPO_4$, 1 mM ATP and 10 µCi [^{32}P]γATP. The results are very similar to the experiments with the buffer lacking Mg^{2+}. Shown is the mean ± SD, n = 3. Numbers above bars indicate the exact calculated p-values.

Supplementary References:

Baconnais S, Tirouvanziam R, Zahm JM, de Bentzmann S, Péault B, Balossier G, and Puchelle E (1999) Ion composition and rheology of airway liquid from cystic fibrosis fetal tracheal xenografts. *Am. J. Respir. Cell Mol. Biol.* **20:**605–611

S-Fig. S1

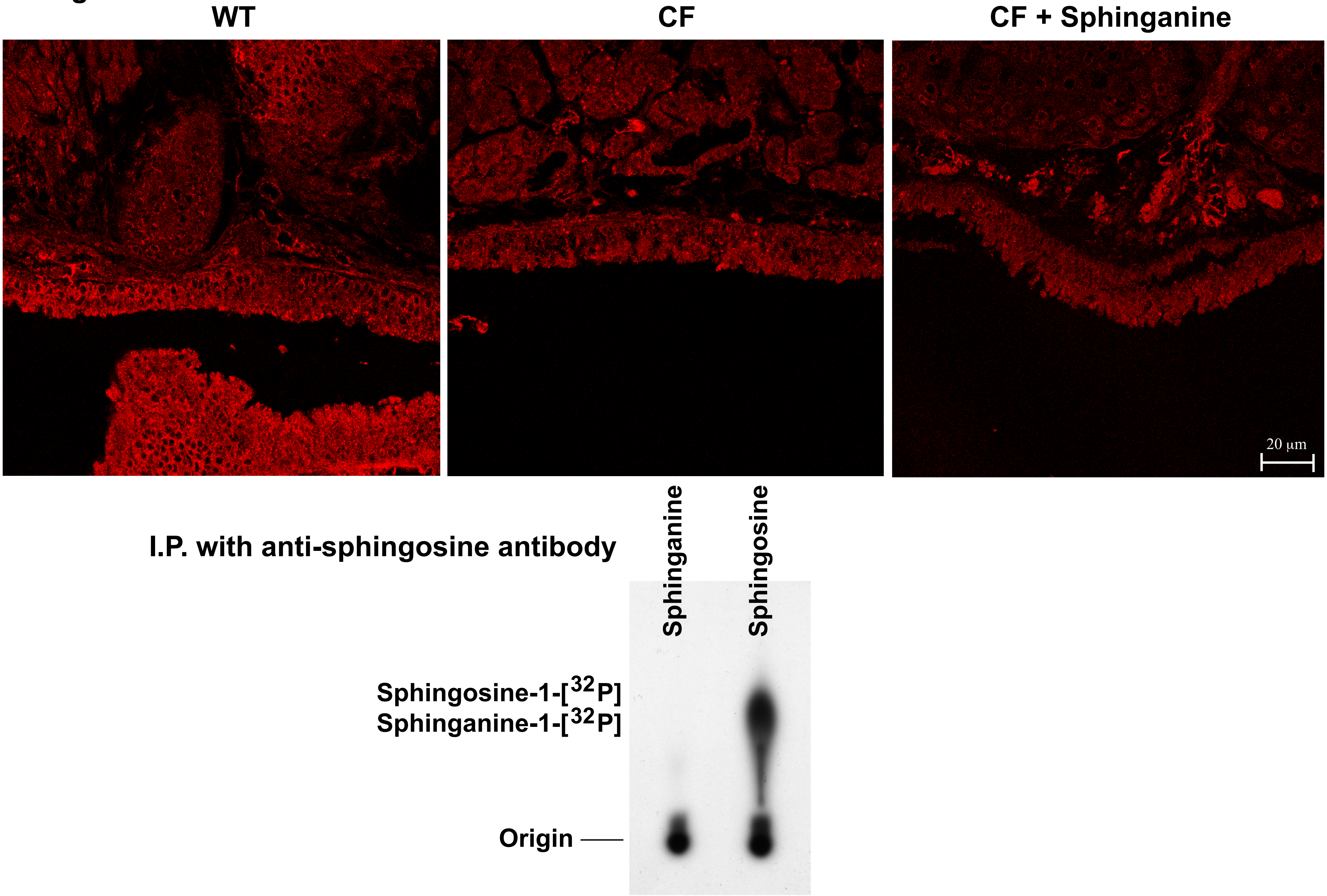
WT
CF
CF + Sphinganine
20 µm
I.P. with anti-sphingosine antibody
Sphinganine
Sphingosine
Sphingosine-1-[32P]
Sphinganine-1-[32P]
Origin

S-Fig. S2

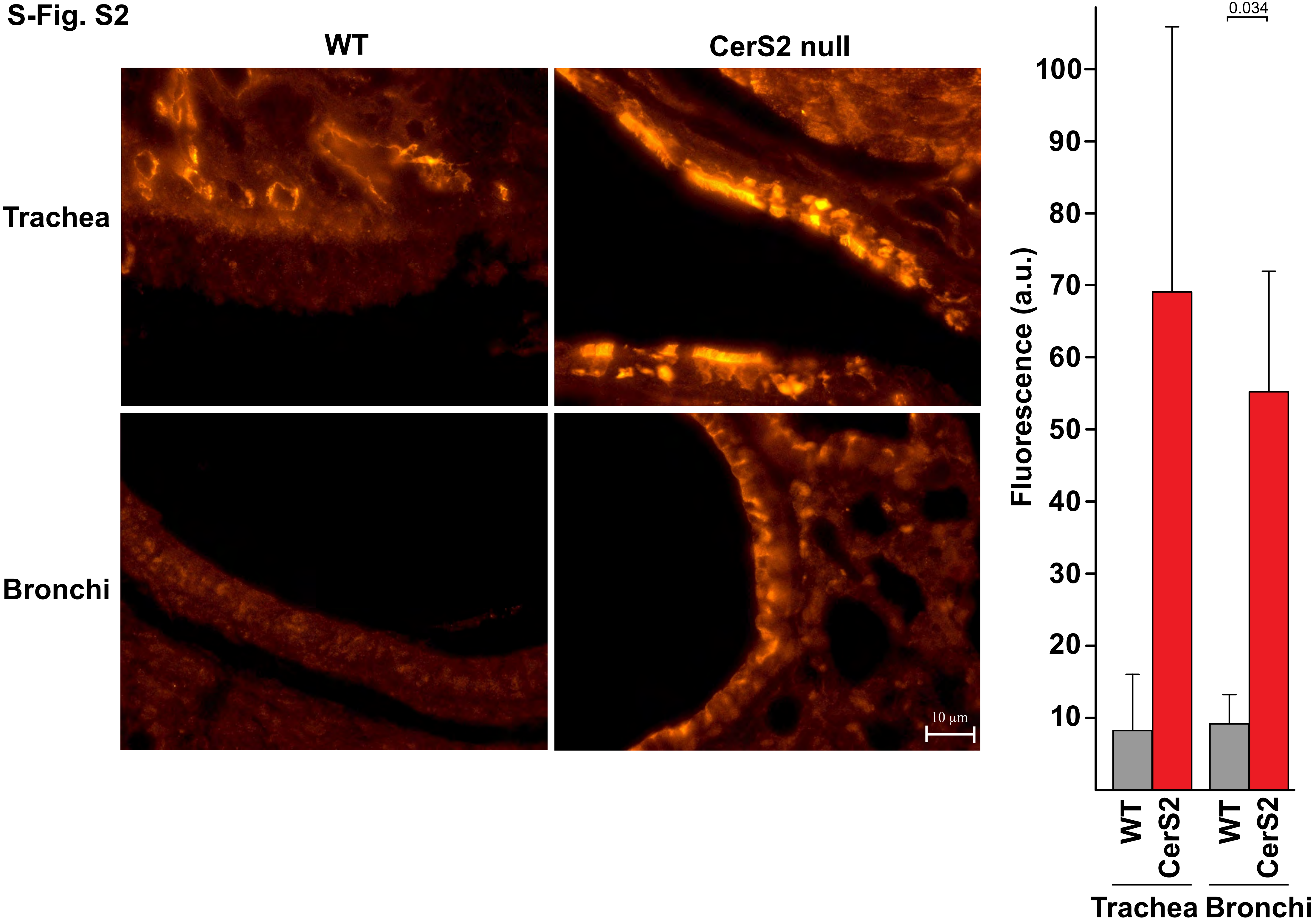
WT
CerS2 null
Trachea
Bronchi
10 µm
0.034
100
90
80
70
60
50
40
30
20
10
Fluorescence (a.u.)
WT
CerS2
WT
CerS2
Trachea
Bronchi

S-Fig. S3A

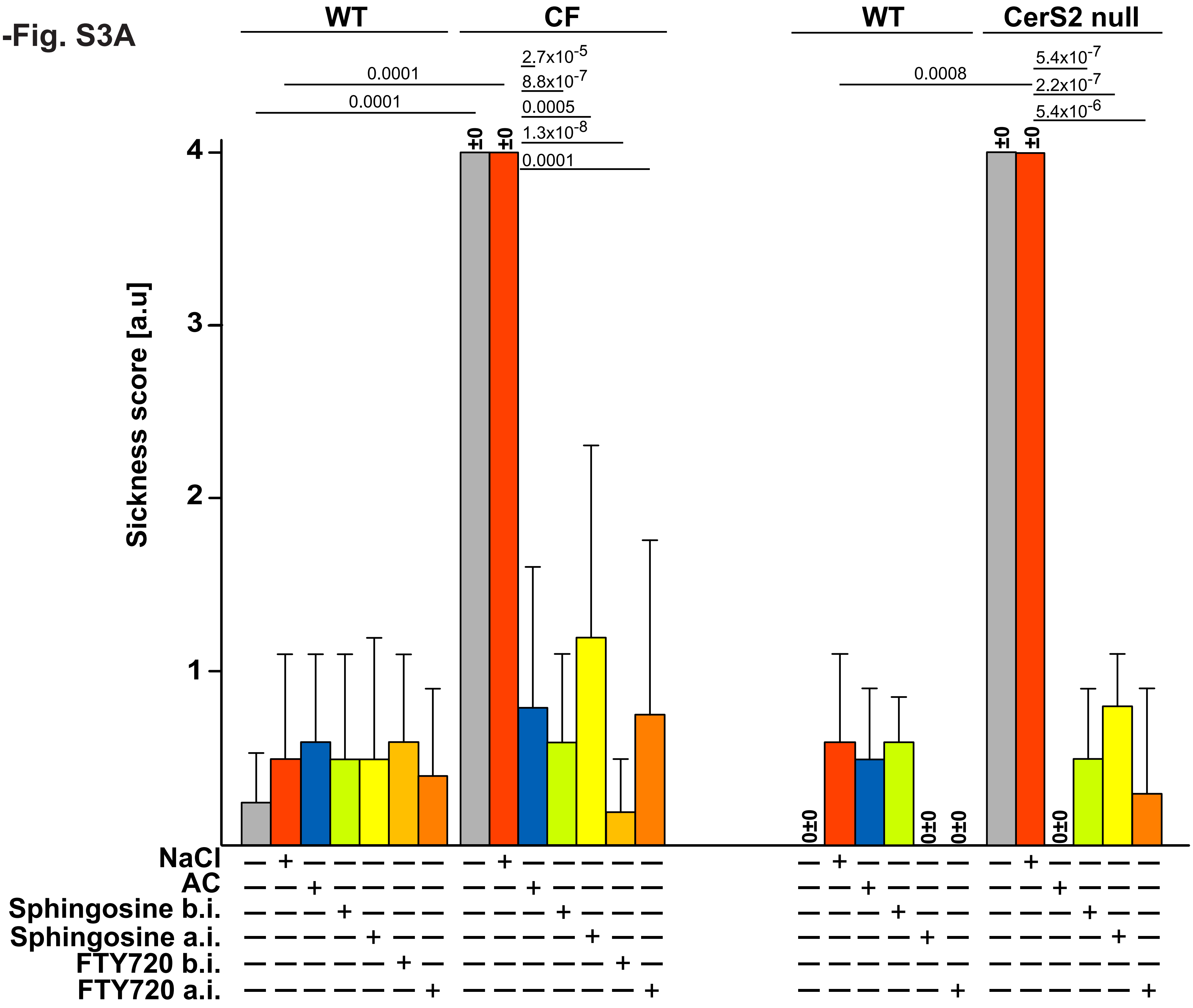

WT
CF
WT
CerS2 null
0.0001
0.0001
2.7x10-5
8.8x10-7
0.0005
1.3x10-8
0.0001
0.0008
5.4x10-7
2.2x10-7
5.4x10-6
±0
0±0
Sickness score [a.u]
4
3
2
1
NaCl
AC
Sphingosine b.i.
Sphingosine a.i.
FTY720 b.i.
FTY720 a.i.

S-Fig. S3B

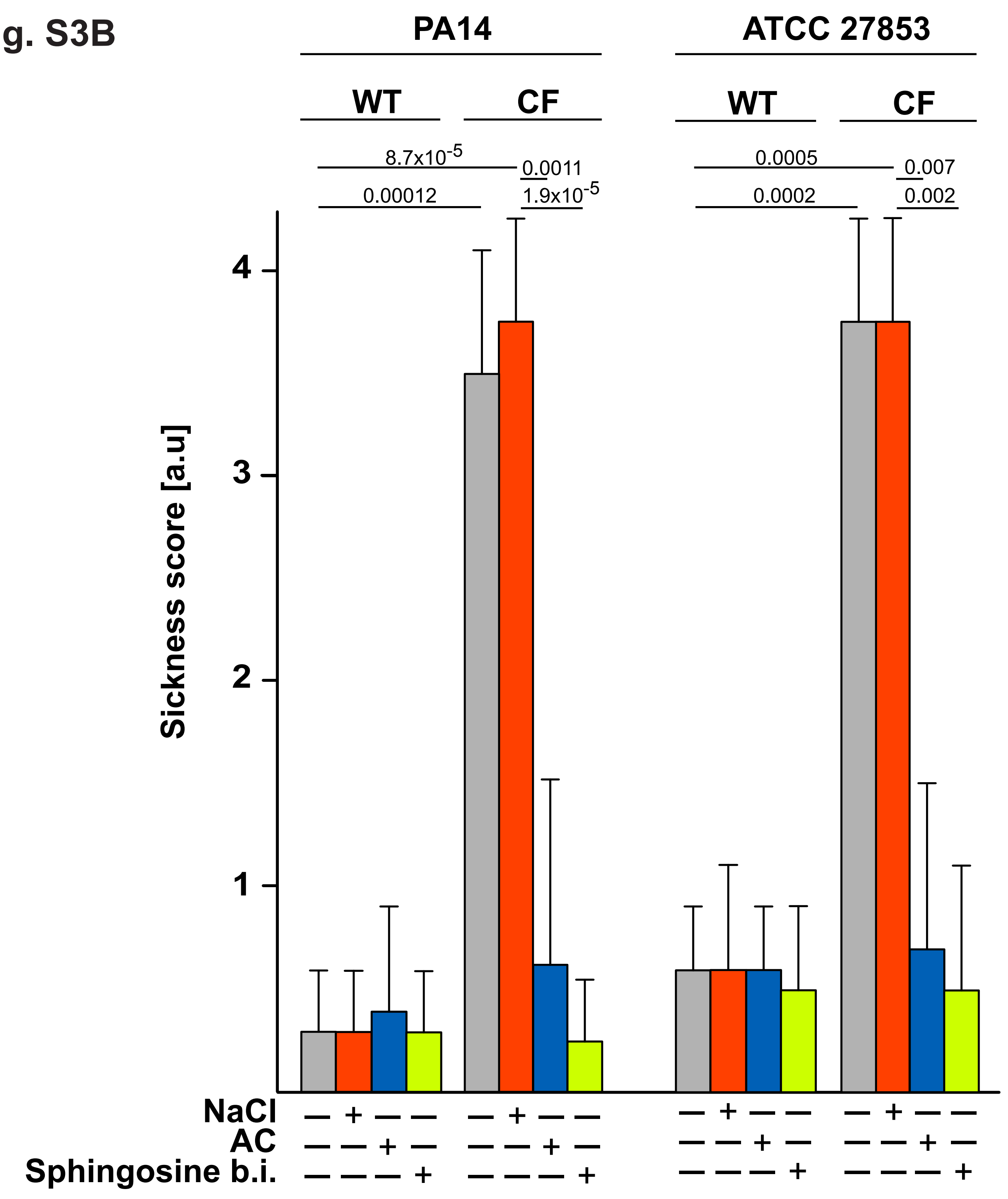

PA14
ATCC 27853
WT
CF
WT
CF
8.7x10-5
0.0011
0.00012
1.9x10-5
0.0005
0.007
0.0002
0.002
Sickness score [a.u]
4
3
2
1
NaCl
AC
Sphingosine b.i.

S-Fig. S4

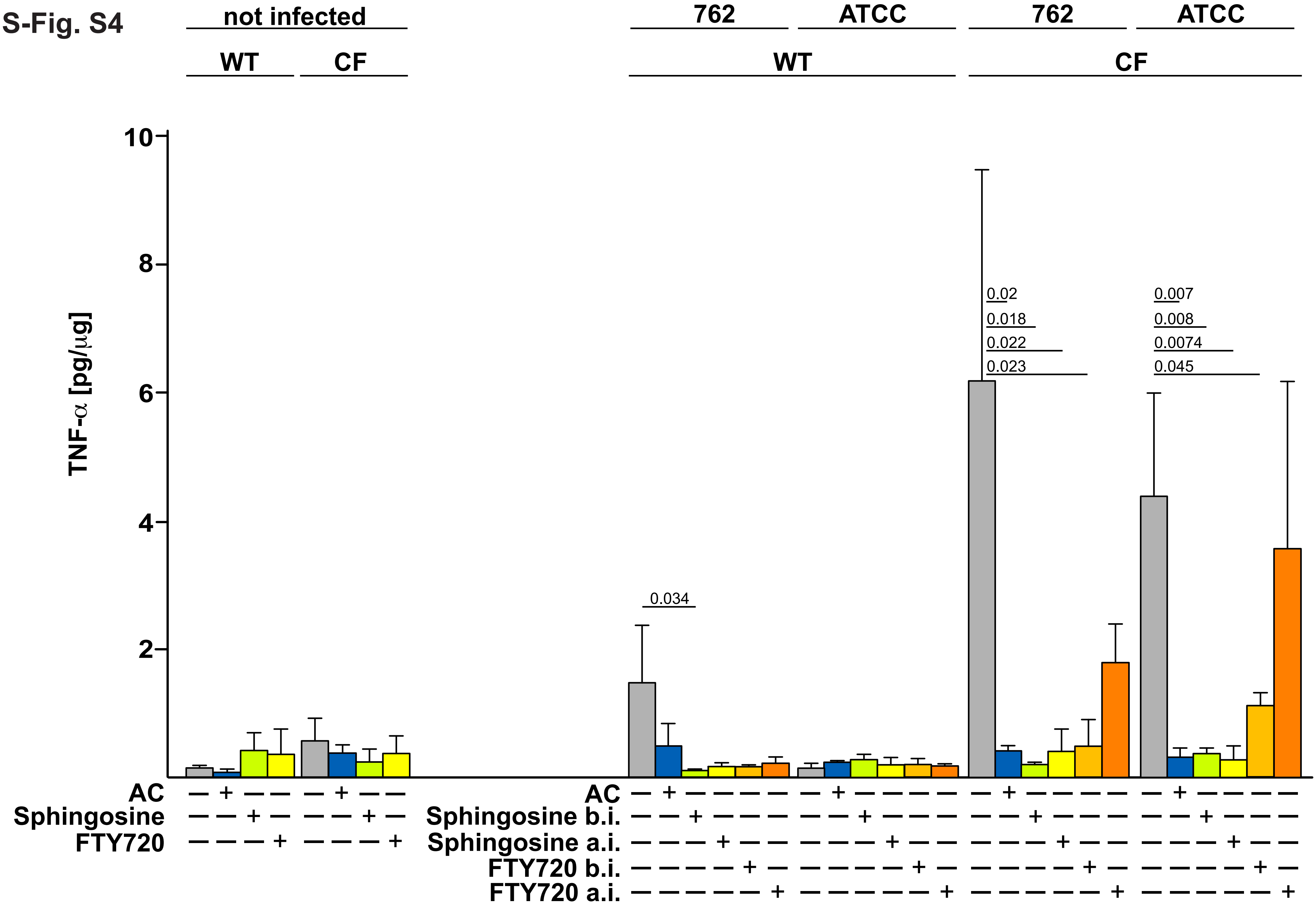
not infected
WT
CF
762
ATCC
762
ATCC
WT
CF
TNF-α [pg/μg]
10
8
6
4
2
0.034
0.02
0.018
0.022
0.023
0.007
0.008
0.0074
0.045
AC
Sphingosine
FTY720
AC
Sphingosine b.i.
Sphingosine a.i.
FTY720 b.i.
FTY720 a.i.

S-Fig. S5A

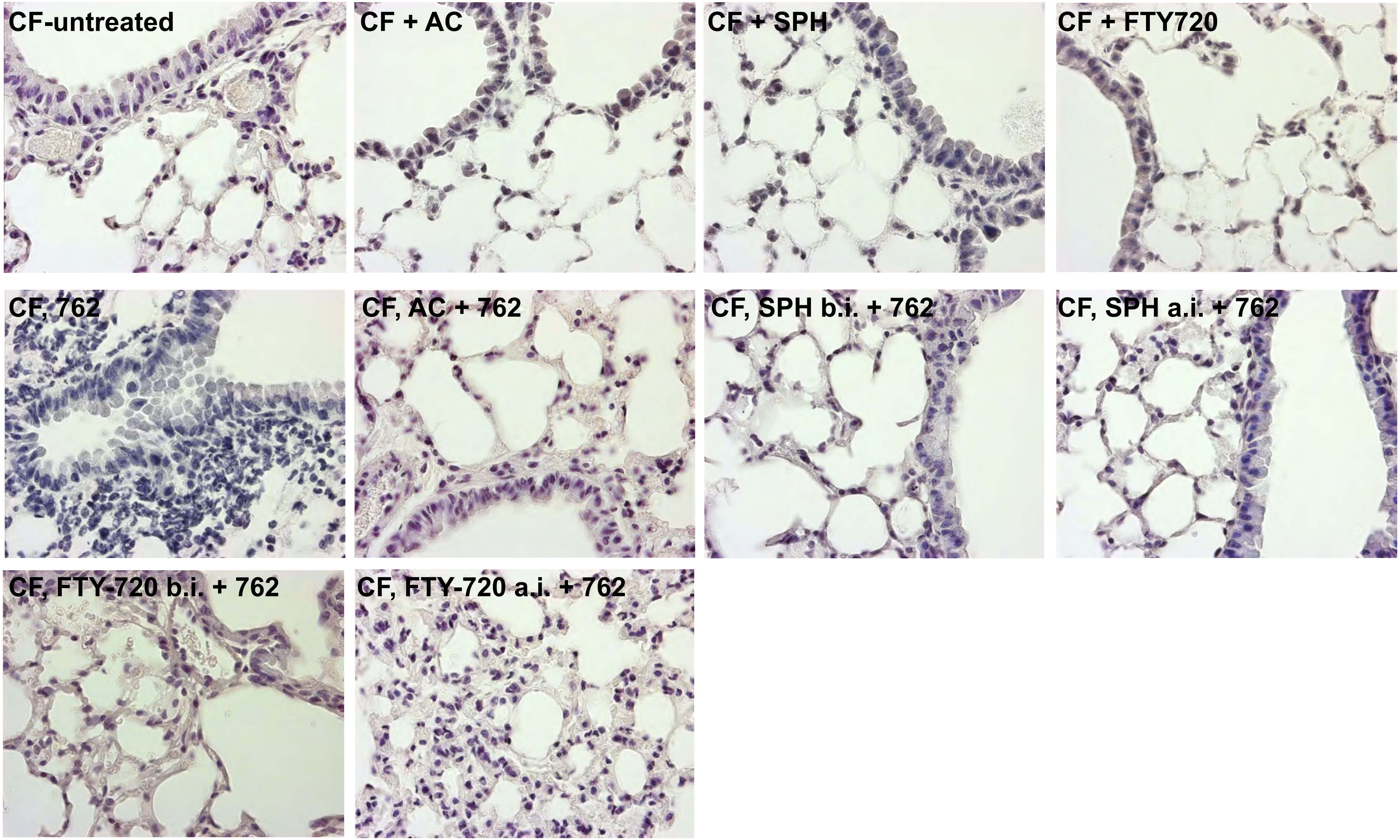
CF-untreated
CF + AC
CF + SPH
CF + FTY720
CF, 762
CF, AC + 762
CF, SPH b.i. + 762
CF, SPH a.i. + 762
CF, FTY-720 b.i. + 762
CF, FTY-720 a.i. + 762

S-Fig. S5B

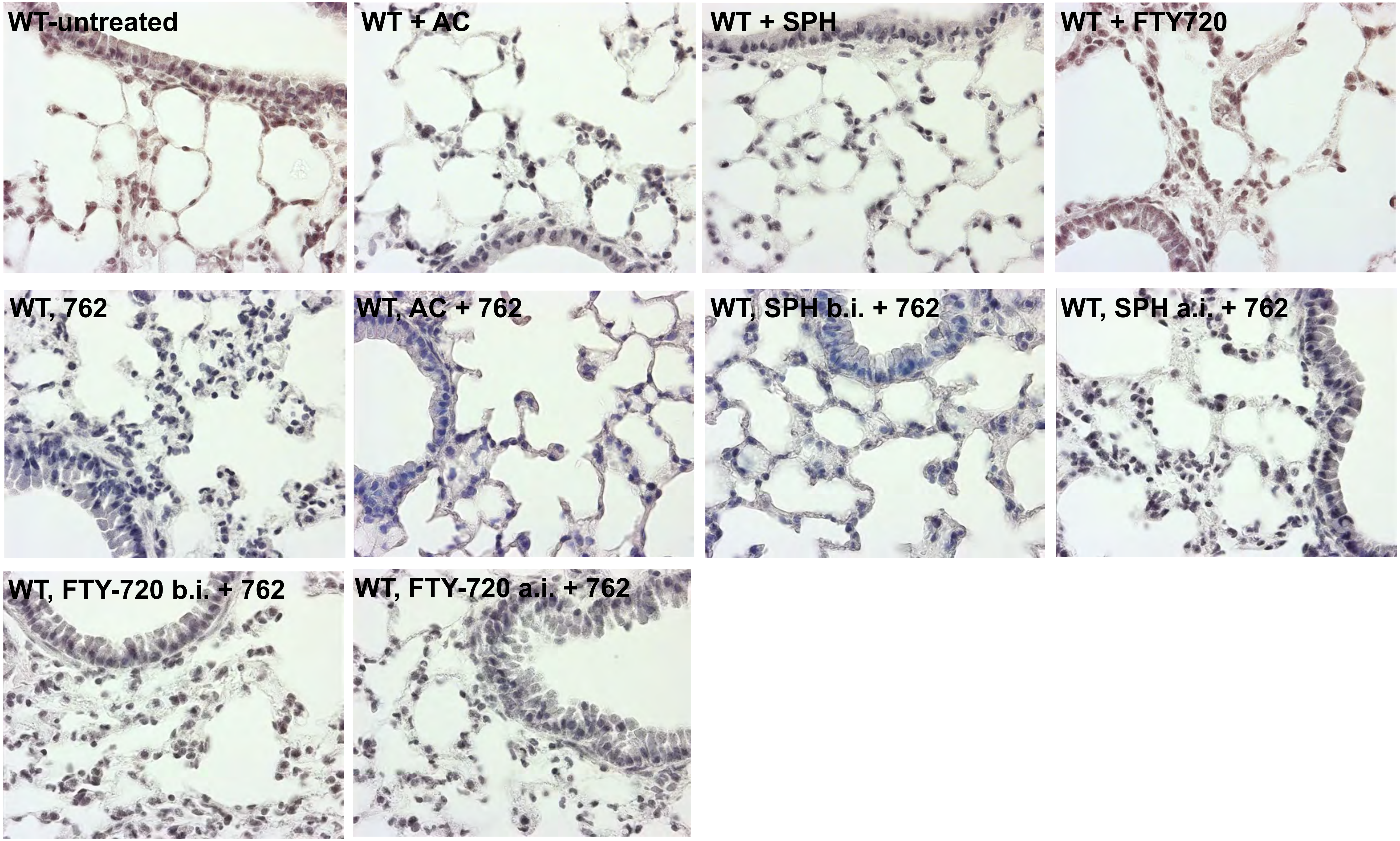
WT-untreated
WT + AC
WT + SPH
WT + FTY720
WT, 762
WT, AC + 762
WT, SPH b.i. + 762
WT, SPH a.i. + 762
WT, FTY-720 b.i. + 762
WT, FTY-720 a.i. + 762

S-Fig. S5C

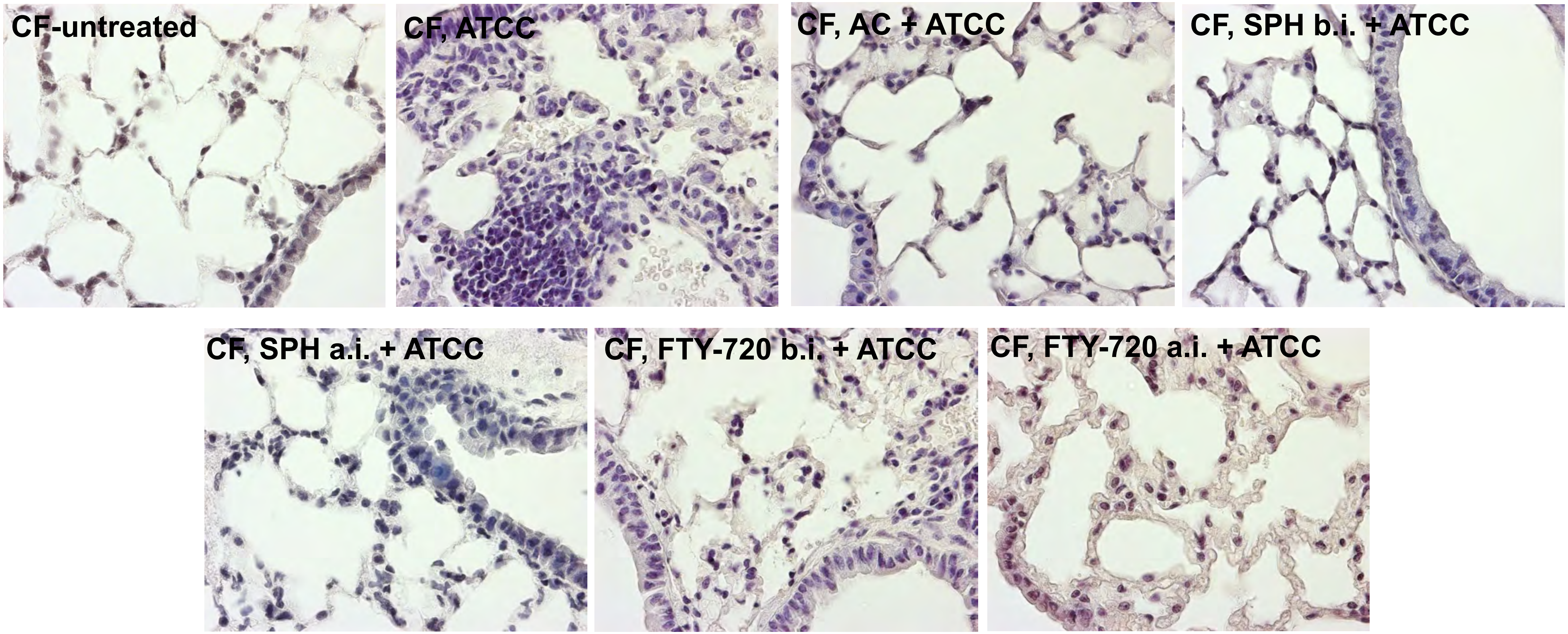
CF-untreated
CF, ATCC
CF, AC + ATCC
CF, SPH b.i. + ATCC
CF, SPH a.i. + ATCC
CF, FTY-720 b.i. + ATCC
CF, FTY-720 a.i. + ATCC

S-Fig. S5D

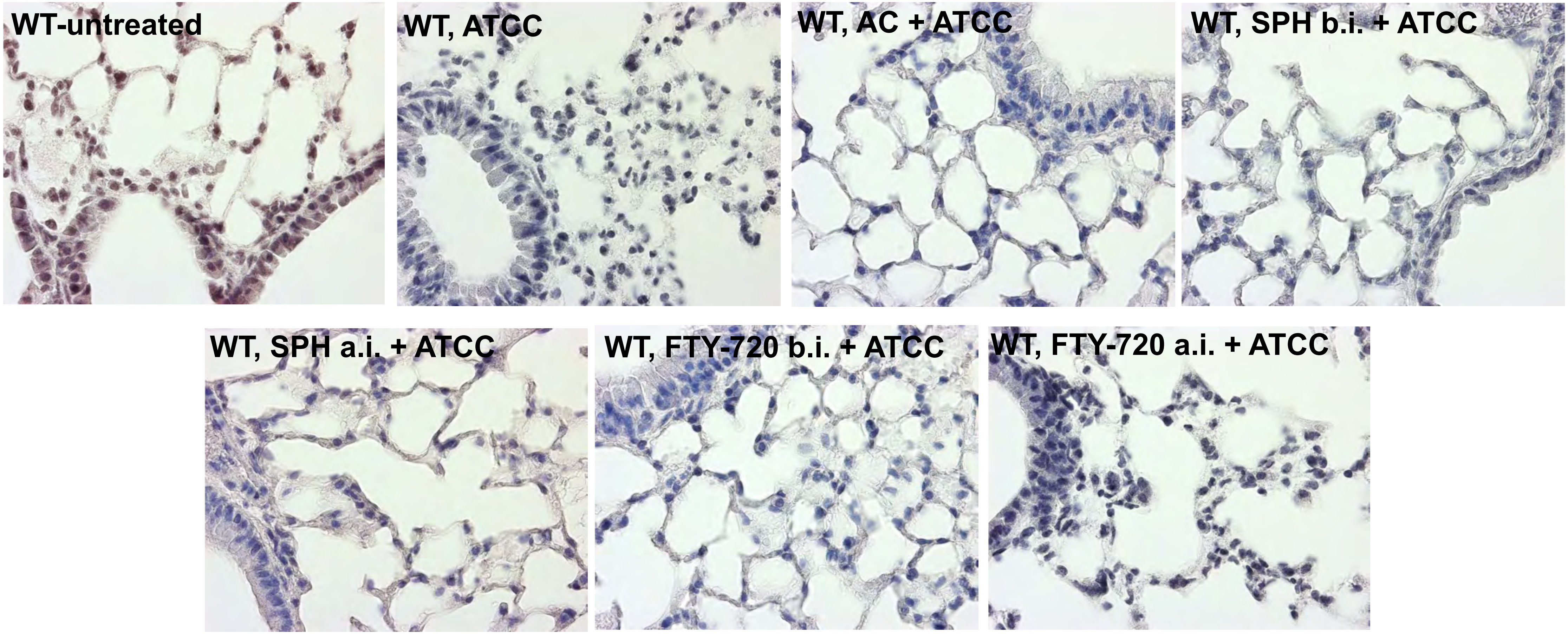
WT-untreated
WT, ATCC
WT, AC + ATCC
WT, SPH b.i. + ATCC
WT, SPH a.i. + ATCC
WT, FTY-720 b.i. + ATCC
WT, FTY-720 a.i. + ATCC

S-Fig. S5E

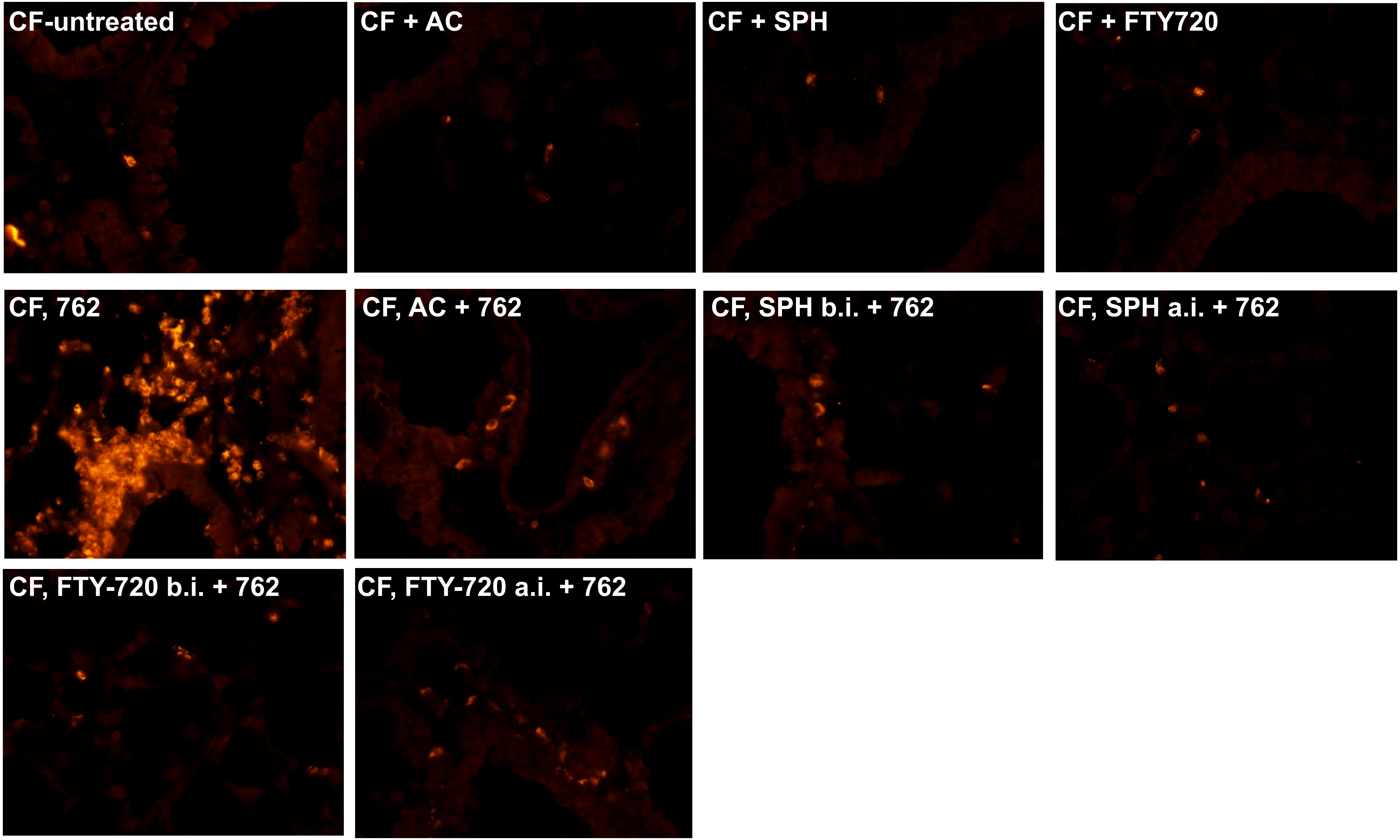
CF-untreated
CF + AC
CF + SPH
CF + FTY720
CF, 762
CF, AC + 762
CF, SPH b.i. + 762
CF, SPH a.i. + 762
CF, FTY-720 b.i. + 762
CF, FTY-720 a.i. + 762

S-Fig. S5F

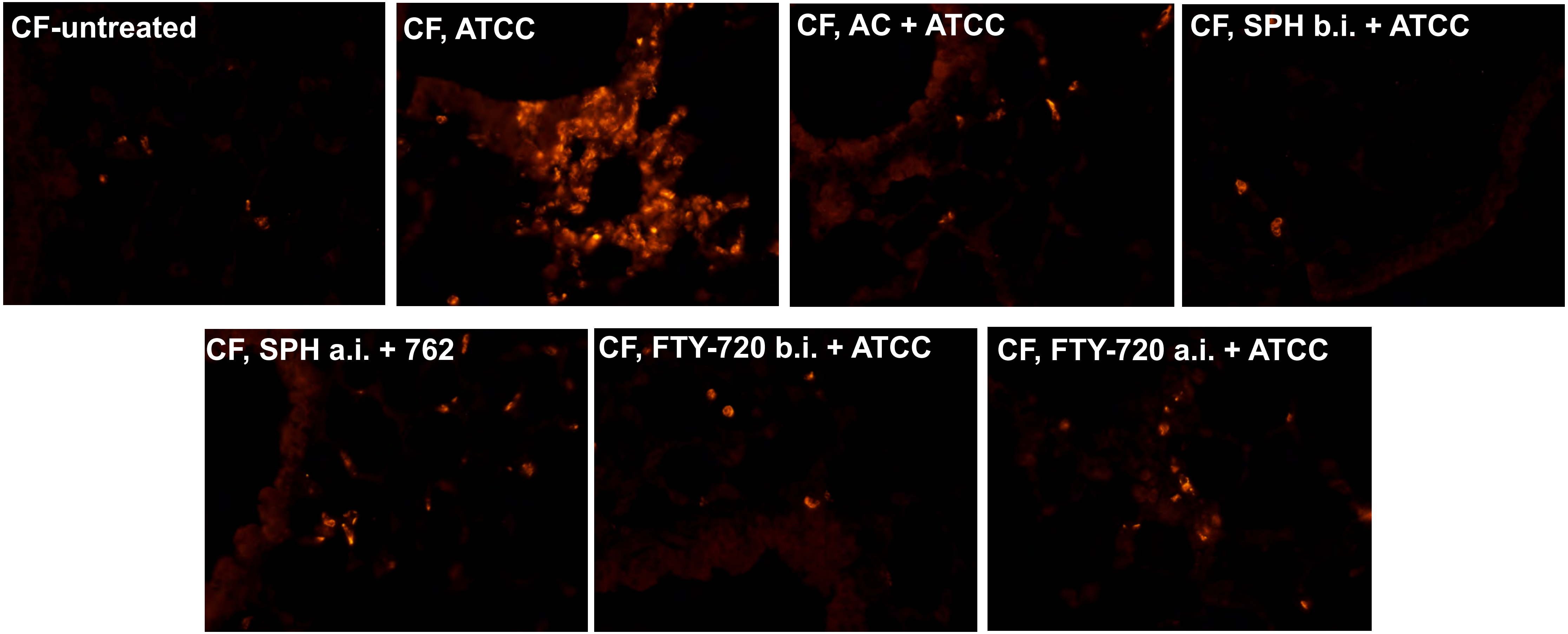
CF-untreated
CF, ATCC
CF, AC + ATCC
CF, SPH b.i. + ATCC
CF, SPH a.i. + 762
CF, FTY-720 b.i. + ATCC
CF, FTY-720 a.i. + ATCC

S-Fig. S5G

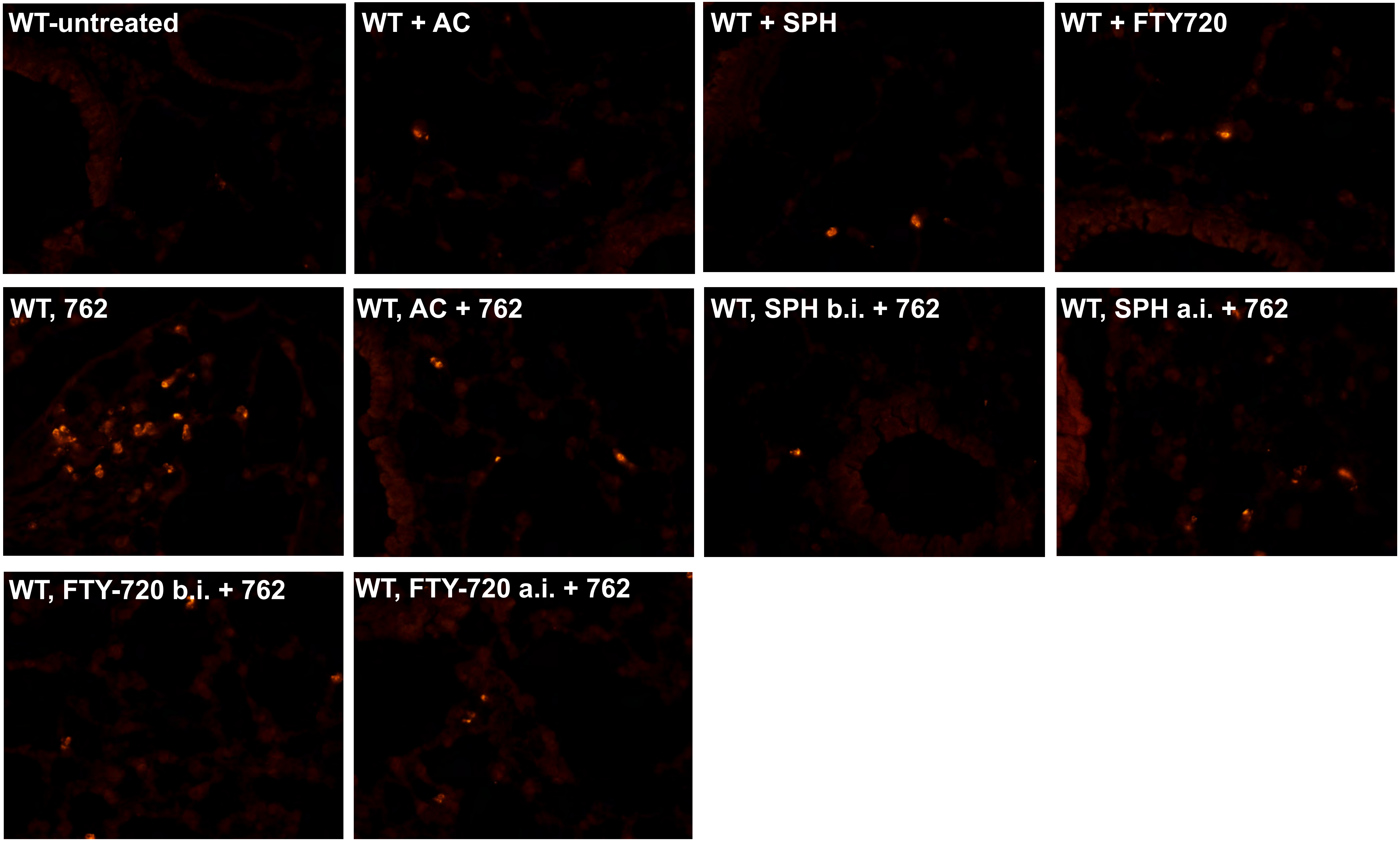
WT-untreated
WT + AC
WT + SPH
WT + FTY720
WT, 762
WT, AC + 762
WT, SPH b.i. + 762
WT, SPH a.i. + 762
WT, FTY-720 b.i. + 762
WT, FTY-720 a.i. + 762

S-Fig. S5H

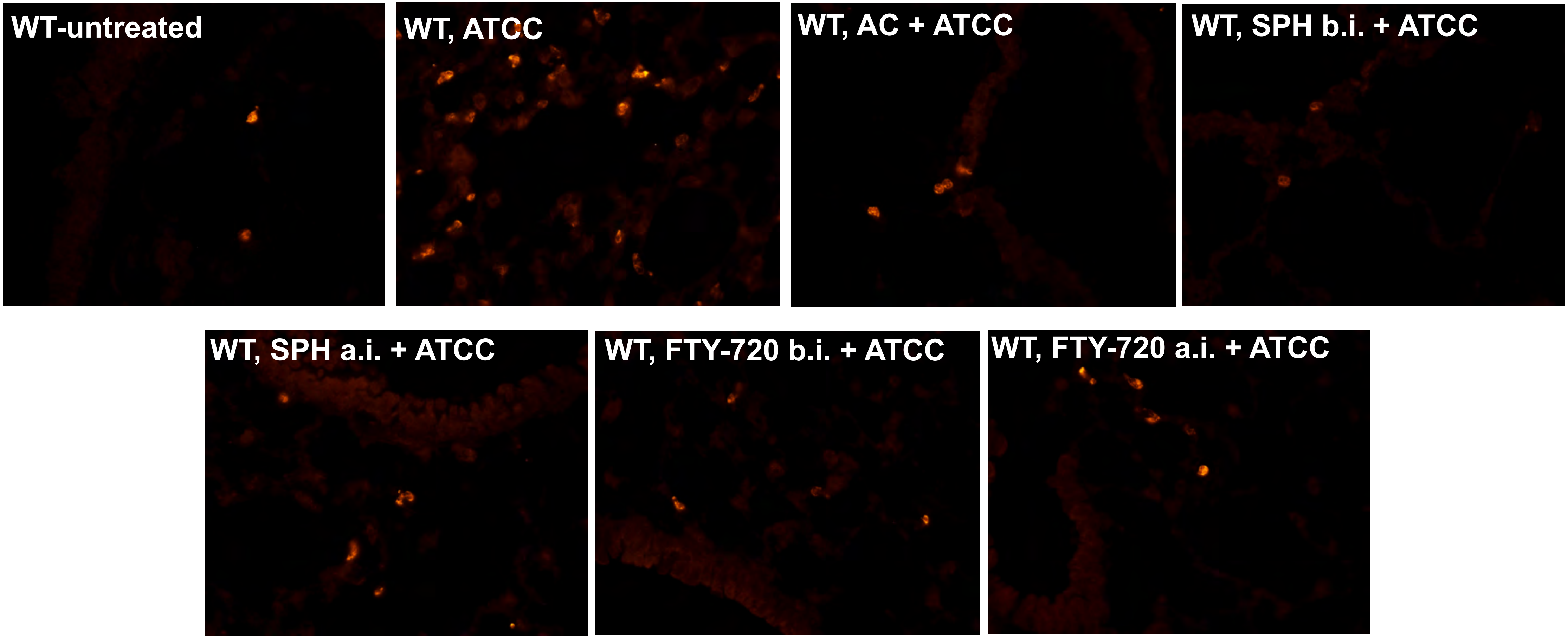
WT-untreated
WT, ATCC
WT, AC + ATCC
WT, SPH b.i. + ATCC
WT, SPH a.i. + ATCC
WT, FTY-720 b.i. + ATCC
WT, FTY-720 a.i. + ATCC

S-Fig. S6A

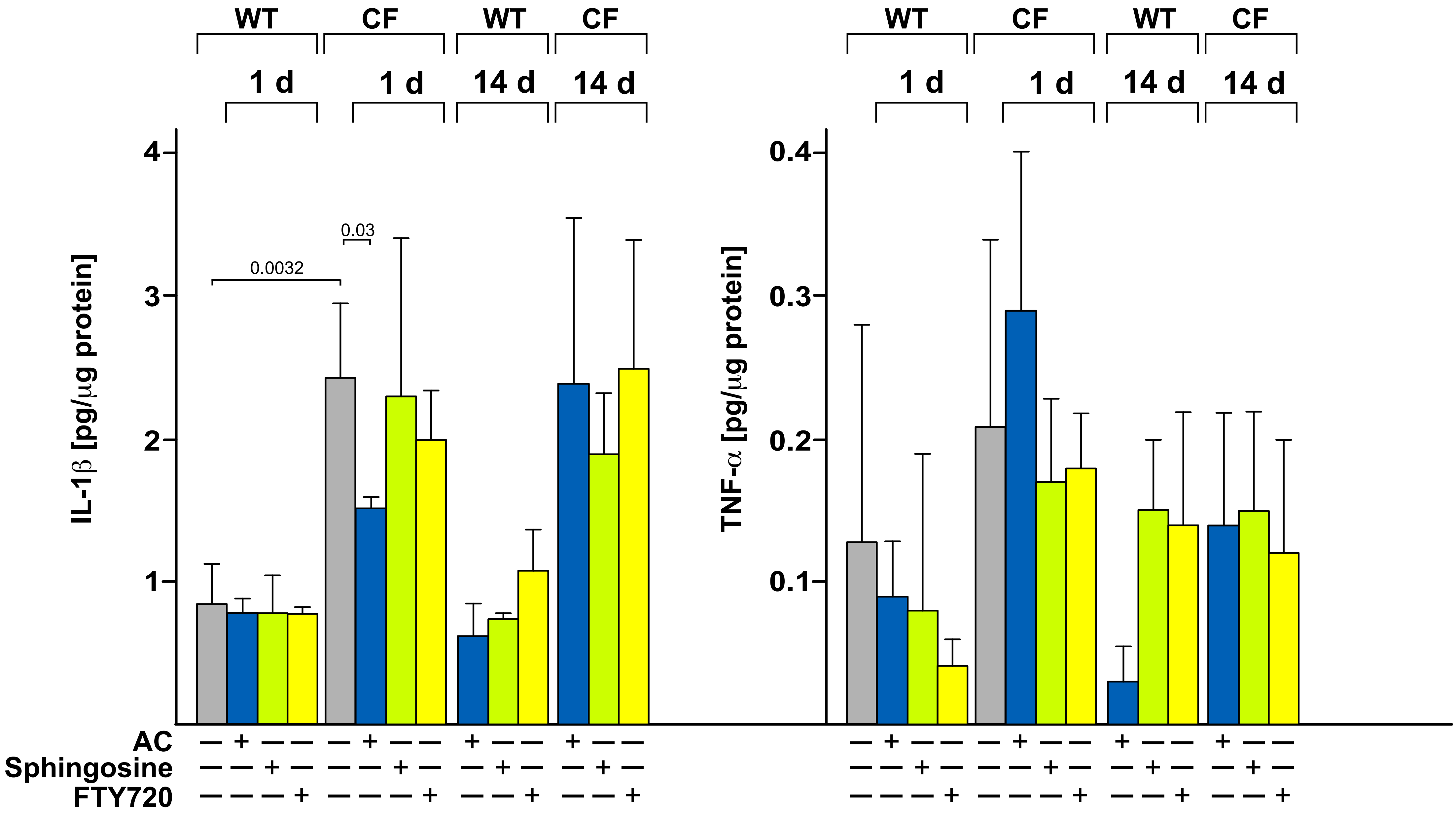
WT
CF
WT
CF
1 d
1 d
14 d
14 d
IL-1β [pg/μg protein]
0.0032
0.03
4
3
2
1
TNF-α [pg/μg protein]
0.4
0.3
0.2
0.1
AC
Sphingosine
FTY720

S-Fig. S6B

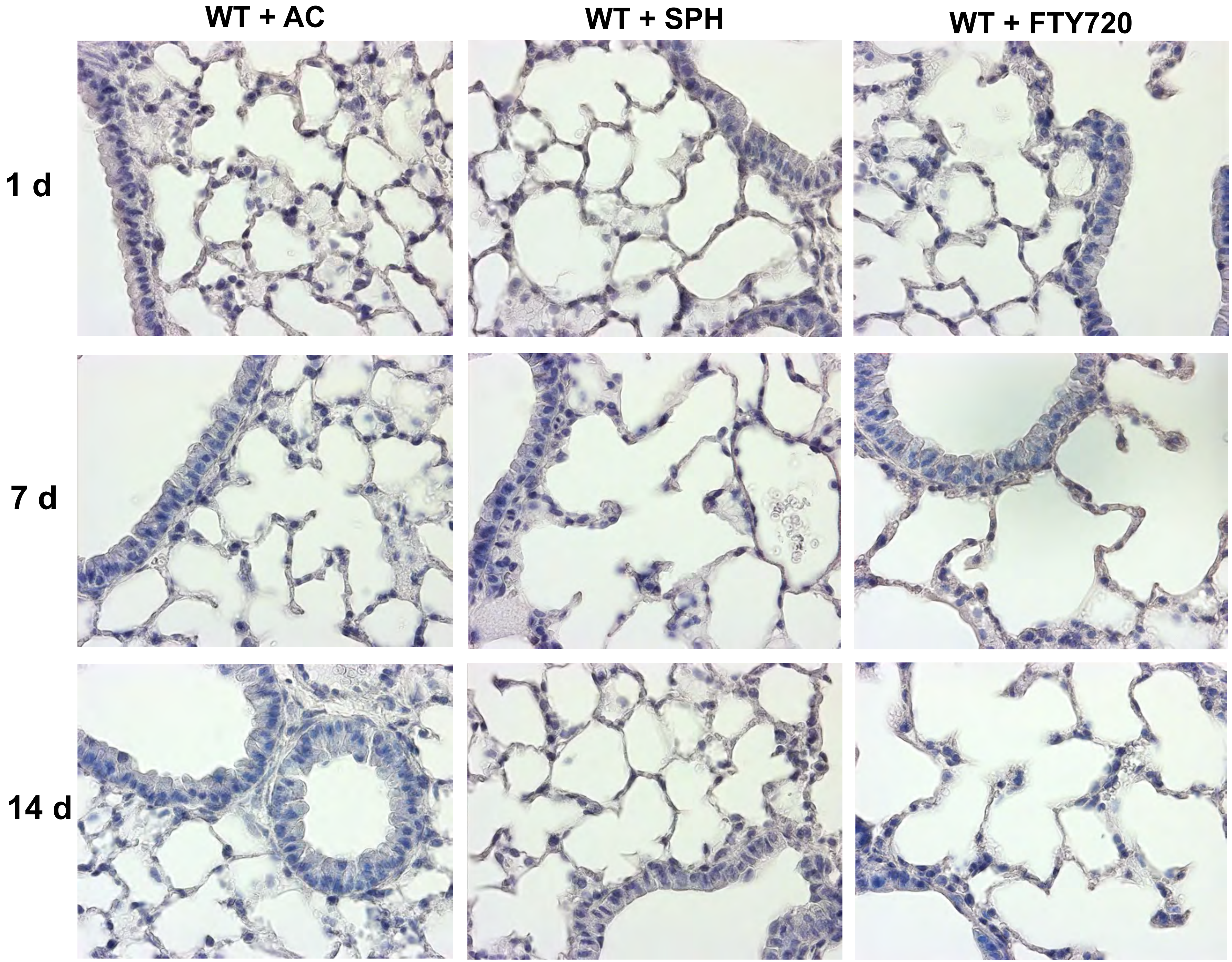

S-Fig. S6C

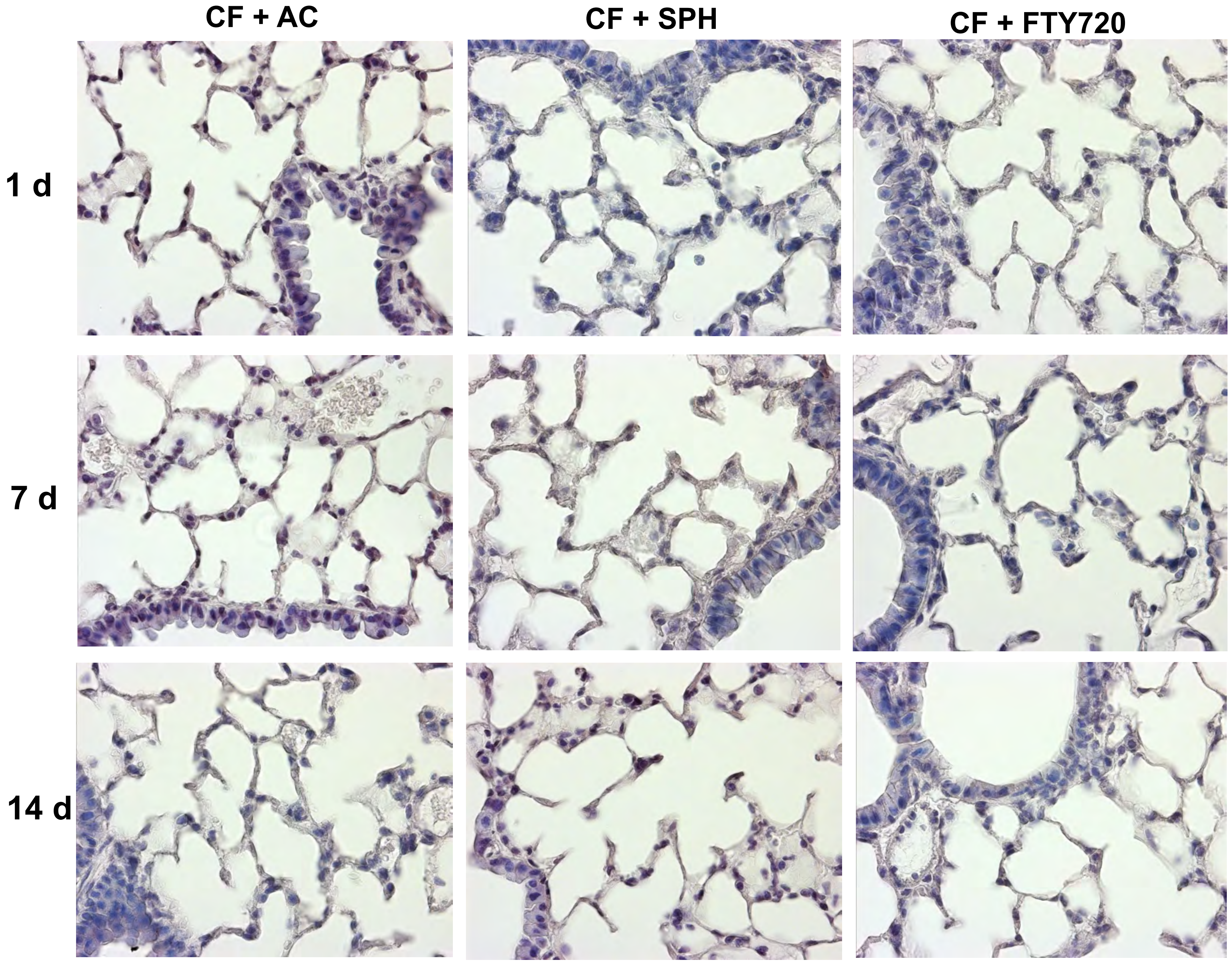

S-Fig. S7

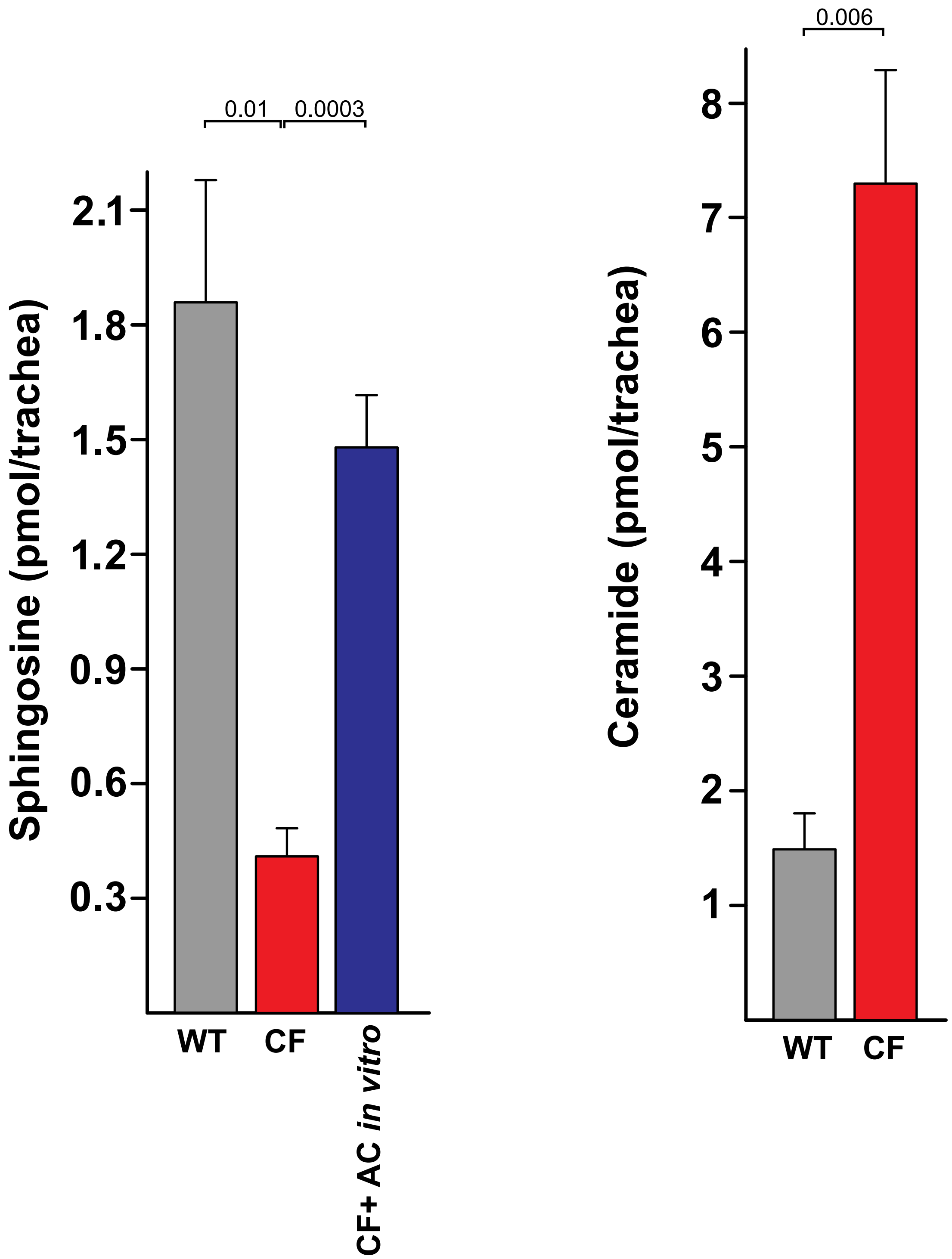
0.01
0.0003
Sphingosine (pmol/trachea)
2.1
1.8
1.5
1.2
0.9
0.6
0.3
WT
CF
CF+ AC *in vitro*
0.006
Ceramide (pmol/trachea)
8
7
6
5
4
3
2
1
WT
CF
